# The EMPEROR-Reduced trial: SGLT2 inhibitors for heart failure get more support

**DOI:** 10.21542/gcsp.2020.31

**Published:** 2020-12-31

**Authors:** Kerolos Wagdy

**Affiliations:** 1 Aswan Heart Center, Division of Cardiology, Aswan, Egypt

## Introduction

**Figure 1.**
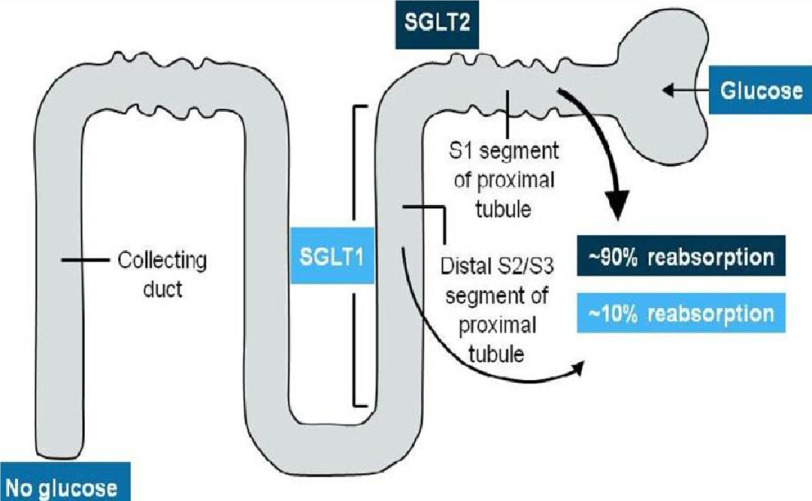
Mechanism and site of action of SGLT2 inhibitors.

Sodium-glucose co-transporter 2 (SGLT2) inhibitors are a relatively new class of antihyperglycemic medication that are well established for the management of type-2 diabetes mellitus (DM).^1^ They have a unique mechanism of action that targets the kidneys through inhibition of 90% of glucose reabsorption[Figure 1].

SGLT2 inhibitors are double-edged weapons. Beside their antihyperglycemic role, they also have an emerging cardiovascular protective role and are associated with natriuresis and diuresis, weight reduction, blood pressure reduction, reducing diabetes-related ventricular remodeling, and other potential cardiovascular benefits.^2^

Three approved agents of the SGLT2 inhibitors from the FDA are empagliflozin, canagliflozin and dapagliflozin. The most common side effects of SGLT2 inhibitors are polyurea, volume depletion (due to it osmotic effect) and genitourinary infections (as it causes high glucose in the urine). However, the serious infections, that may reach to urosepsis or pyelonephritis, are very rare. Other rare side effects are euoglycemic diabetic ketoacidosis and hypoglycemia particularly when is concurrently prescribed with insulin or sulphonylureas.

Over the last five years, SGLT2 inhibitors have had an exciting and growing role in cardiovascular (CV) protection that was reported in three landmark trials (EMPA-REG, CANAVAS and DECLARE-TIMI 58). For the first time in the history of type-2 DM, there are data that indicates CV benefits from the use of glucose-lowering drugs in patients with CV disease or at CV risk.^3^

The EMPA-REG OUTCOME trial demonstrated that empagliflozin reduces mortality, hospitalization for heart failure and renal events in type-2 diabetic patients with cardiovascular risk.^4^ Moreover, the CANVAS trial for canagliflozin and DECLARE-TIMI 58 trial for dapagliflozin, showed similar beneficial cardiovascular and renal effects in diabetic patients at cardiovascular risk.^5,6,7^

Recently, the DAPA-HF trial showed that dapagliflozin reduced cardiovascular deaths and heart failure events when compared to placebo in patients already diagnosed to have heart failure with reduced ejection fraction (HFrEF). The primary composite outcome of worsening heart failure or death from cardiovascular causes was lower in the dapagliflozin group (16.3%) compared with 21.2% in the placebo group (hazard ratio, 0.74; P<0.001).^8^ Recently, the FDA approved its use in the heart failure management, based on DAPA-HF trial.

Further evidence is needed to support the role of SGLT2 inhibitors in the management of heart failure and particularly for agents other than dapagliflozin. Therefore, the EMPEROR-Reduced trial was conducted to assess the efficacy of empagliflozin in management of heart failure with reduced ejection fraction, regardless of DM.

## The study

Empagliflozin Outcome Trial in Patients with Chronic Heart Failure and a Reduced Ejection Fraction (EMPEROR-Reduced) trial is an international multicenter randomized placebo-controlled trial with double-blinded design. The study was published in *New England Journal of Medicine* (NEJM) in August 2020.^9^ The study enrolled 3730 patients with symptomatic and stable heart failure with reduced ejection fraction (EF ≤ 40%) to assess the efficacy and safety of empagliflozin in patients, regardless of diabetes mellitus.

One arm of patients received empagliflozin 10 mg once daily while another arm received placebo, in addition to optimized guideline directed medical therapy for heart failure in both groups. The study was intended to enroll patients with increased risk of serious HF events; by enrolling patients with severely impaired left ventricular (LV) function (EF ≤30%) or patients with moderately impaired LV function but have history of HF hospitalization or significantly high NT-Pro BNP.

**Figure 2.**
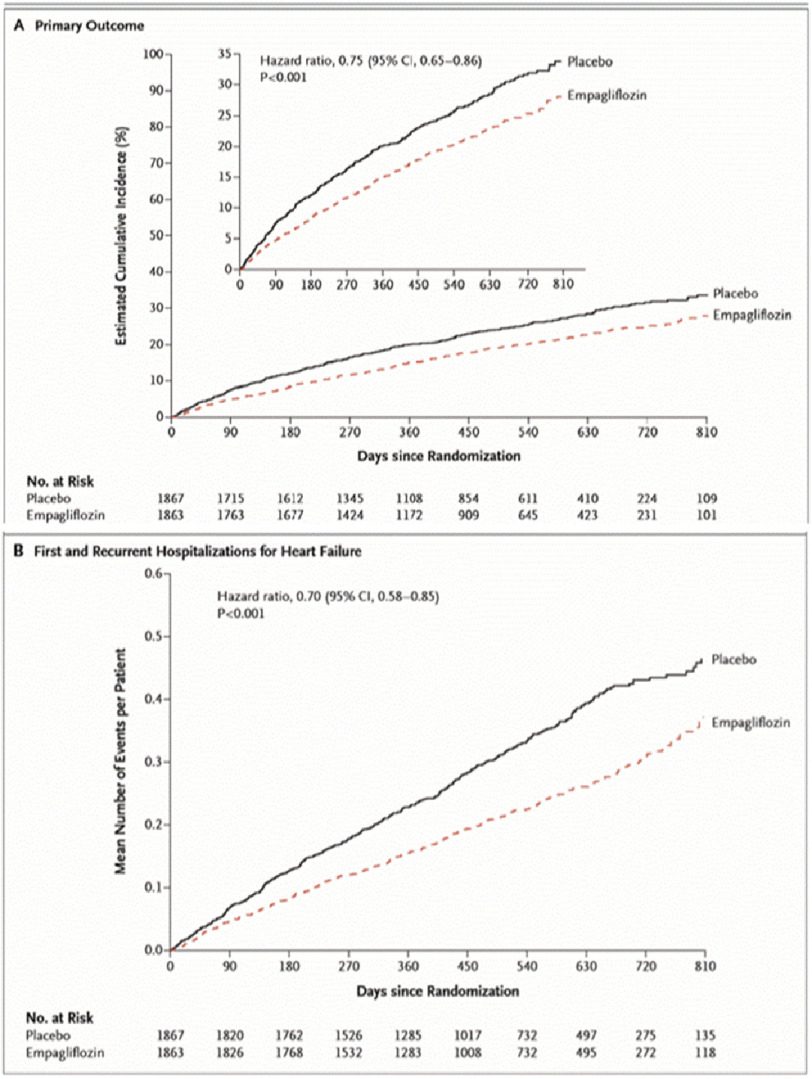
Primary outcomes between empagliflozin and placebo groups.

Patients were followed up every 2–3 months (for median duration of 16 months) to assess outcomes, adverse events, functional capacity assessment, NT-Pro BNP and renal function. The study showed a significant reduction of the combined primary endpoints (cardiovascular death and hear failure hospitalization) in the Empagliflozin group compared to placebo (19.4% vs. 24.7% respectively; hazard ratio 0.76, 95% CI [0.67–0.87]; *P* < 0.0001) (Figure 2).

The results were consistent in patients with or without diabetes and among those were on Angiotensin receptor/ Neprilysin inhibitor (ARNI) or not. Additionally; empagliflozin reduced the total number of HF hospitalizations (hazard ratio 0.67, 95% CI [0.50–0.90], *P* = 0.008). The empagliflozin group had a slower rate of decline in renal function (estimated by GFR). Moreover, patients assigned to empagliflozin were 20–40% more likely to experience an improvement in NYHA functional class and were 20–40% less likely to experience worsening of NYHA functional class. Empagliflozin arm showed higher uncomplicated urinary tract infection.

## Discussion

Heart failure remains a challenging health problem with high morbidity and mortality despite sustained advances in its management with different pharmacological, device and surgical options.

Over the few last years, many variant classes of drugs have been developed (ARNI, Vericiguat and SGLT2 inhibitors) that have an emerging role in the management of heart failure with reduced ejection fraction through different mechanisms rather than the classical renin-angiotensin-aldosterone blockage mechanism.

The EMPEROR-Reduced trial confirmed the results of DAPA-HF, that SGLT2 inhibitors reduce cardiovascular death and heart failure hospitalization compared to placebo. It showed this effect is a class effect (SGLT2 inhibitors) limited to dapagliflozin (drug effect). Additionally, it demonstrated this beneficial effect in patients with severe heart failure, who are at higher risk of adverse events and higher mortality, as more than 70% of the enrolled patients had EF ≤30% (as DAPA-HF enrolled patients with mild and moderate heart failure).

The promising results of the EMPEROR-Reduced trial and the raising role of empagliflozin in heart failure with reduced ejection fraction encouraged the investigators to conduct an EMPEROR-Preserved trial to assess the efficacy and safety of empagliflozin in heart failure with preserved ejection fraction. That trial is still ongoing (https://clinicaltrials.gov/ct2/show/NCT03057951).

## What we have learnt?

In the EMPEROR-Reduced trial, SGLT2 inhibitors have gotten more evidence for use in heart failure management. Empagliflozin became the second agent of SGLT2 inhibitors (after dapagliflozin) that produces a significant improvement in cardiovascular death and heart failure hospitalization in patients with severe heart failure, whether diabetic or not. Additionally, it improves the functional capacity and quality of life.

The EMPEROR-Reduced trial reinforced the results of DAPA-HF trial and confirmed the class effect of SGLT2 inhibitors in HFrEF patients, rather than drug effect (limited to dapagliflozin). The trial showed an early separation of the curves between the placebo and empagliflozin arms indicating the early benefit. Moreover, the trial yielded significant renal protection with empagliflozin in heart failure patients who are prone to worsening renal function.

Regarding safety, serious adverse events related to cardiac disorders or worsening renal function were lower in the empagliflozin group.
